# Identifying developmental vulnerability through linear growth screening: a UK cross-sectional study

**DOI:** 10.1136/bmjph-2025-004567

**Published:** 2026-06-23

**Authors:** Michael Papasavva, Joanna Orr, Isabella Cordani, Lola Oloko, Celes Hutchinson, Christopher Norton, Kaur Kamaljit, Joseph Freer, Robert Walton, Helen L Storr, Melanie Smuk, Andrew J Prendergast

**Affiliations:** 1 Faculty of Medicine and Dentistry, Queen Mary University of London, London, UK; 2 Tower Hamlets GP Care Group CIC, London, UK

**Keywords:** Humans, Public Health, Sociodemographic Factors

## Abstract

**Introduction:**

Linear growth in early childhood is routinely measured in the UK, but it is unclear whether indicators of linear growth faltering can flag children with neurodevelopmental vulnerability, especially in ethnically diverse urban settings. We tested whether short stature (stunting) or being short for genetic potential identifies children at risk of failing routine developmental screening at age 2–2.5 years.

**Methods:**

We conducted a cross-sectional analysis among 555 children aged 24–30 months (mean 26.0 months, SD 1.5) attending Healthy Child Programme reviews in East London. The sample was ethnically diverse, with substantial proportions of Bangladeshi, White and other minority ethnic groups. Developmental vulnerability was defined as scoring below the Ages and Stages Questionnaire, Third Edition (ASQ-3) cut-off in ≥1 domain. The exposures were stunting (height-for-age z score <−2 SD) and being short for genetic potential, defined as deviation from mid-parental height (DMPH) <−2 SD. Associations were examined using binomial multivariable logistic regression adjusted for age, sex, prematurity, maternal education, ethnicity and household benefits. A subset (n=94) completed the Griffiths III Mental Development Scales (GMDS-III) to validate ASQ-3 classification.

**Results:**

Overall, 141/555 (25.4%) children screened positive for developmental vulnerability on the ASQ-3. Children with developmental vulnerability had significantly lower GMDS-III scaled scores across multiple domains supporting criterion validity. We did not find evidence of an association between stunting and developmental vulnerability (adjusted OR 1.35, 95% CI 0.47 to 3.49). In contrast, children who were short for genetic potential (DMPH <−2 SD) had higher odds of developmental vulnerability (adjusted OR 4.18, 95% CI 1.13 to 14.78).

**Conclusions:**

In this urban UK cohort, deviation from genetic height potential, rather than stunting, identified children at increased risk of developmental vulnerability. Incorporating parental heights into routine growth surveillance could offer a simple trigger for earlier neurodevelopmental review.

**WHAT IS ALREADY KNOWN ON THIS TOPIC**

- Early childhood growth is routinely monitored but is not used to identify children with developmental vulnerability in high-income settings.

**WHAT THIS STUDY ADDS**

- Being short for genetic potential (low deviation from mid-parental height), rather than absolute short stature (stunting), was associated with ~fourfold higher odds of failing Ages and Stages Questionnaire, Third Edition screening for developmental vulnerability at 24–30 months.

**HOW THIS STUDY MIGHT AFFECT RESEARCH, PRACTICE OR POLICY**

- Incorporating parental heights into routine growth checks could help health visitors trigger timely, more detailed neurodevelopmental assessment without introducing a new screening programme.

## Introduction

 Early childhood is a critical period for neurodevelopment, during which biological and environmental exposures shape lifelong cognitive, behavioural and educational outcomes.^1^ Adverse factors in early life, including poverty, limited parental education and preterm birth, are known to undermine children’s developmental potential, particularly in socioeconomically deprived settings.^2^ In low- and middle-income countries, impaired linear growth is well established as a marker of cumulative adversity,^3^ and stunting (height-for-age Z-score (HAZ) <−2) has been associated with poor neurocognitive and educational outcomes.^4^ However, the developmental implications of growth faltering in high-income countries (HICs) require further exploration.^5^ There is growing interest in whether poor linear growth, typically operationalised as low height-for-age or deviation from mid-parental height (DMPH), may similarly reflect early disadvantage and predict developmental vulnerability in HIC contexts. If so, linear growth may offer a practical, affordable and scalable marker to identify children who warrant further developmental screening, particularly in disadvantaged populations.

Tower Hamlets, an inner-city borough in east London, is one of the most socioeconomically bimodal areas in the UK and is home to a large and diverse ethnic population, with a high proportion of households of Bangladeshi ethnicity.^6^ Many children experience household poverty, and limited access to early support services that may impact both physical growth and cognitive development. Despite these risks, there is no formal screening programme for neurodevelopment in the UK, although caregiver-reported assessments are undertaken through the Healthy Child Programme (HCP). The HCP offers structured health, growth and development reviews up to age 2.5 years by health visitors (HVs) or nursery nurses (NNs), but after this age, there is no routine monitoring unless specific concerns are raised.^7^

Emerging evidence suggests there may be a missed opportunity to consider growth and development screening in tandem. Nationally representative analyses have revealed that short stature in England is geographically clustered and closely linked to area-level deprivation.^8^ Further, longitudinal data from the Millennium Cohort Study provided evidence that children with short stature at age 3 years have persistently lower language development scores throughout childhood, even after adjusting for sociodemographic factors.^9^ Qualitative research conducted in east London supports the acceptability among caregivers and feasibility among healthcare workers of integrating child height into developmental screening pathways, particularly when accompanied by appropriate referral systems.^10^

To investigate whether linear growth screening has potential value in a contemporary UK context, we established the Child Growth and Development in East London (CGDEL) study. CGDEL combines caregiver-reported developmental screening using the Ages and Stages Questionnaire (ASQ) with height screening using an automated algorithm at age 2–2.5 years through the HCP. Within the study, we aimed to determine whether screening for linear growth faltering could also identify children who require further developmental assessment. Our rationale was that shared risk factors and mechanistic pathways may underlie linear growth faltering and impaired neurodevelopment. Demonstrating this link would provide evidence for potential dual benefits of a broader growth screening programme in the UK.

## Methods

### Participants

**Figure 1:**
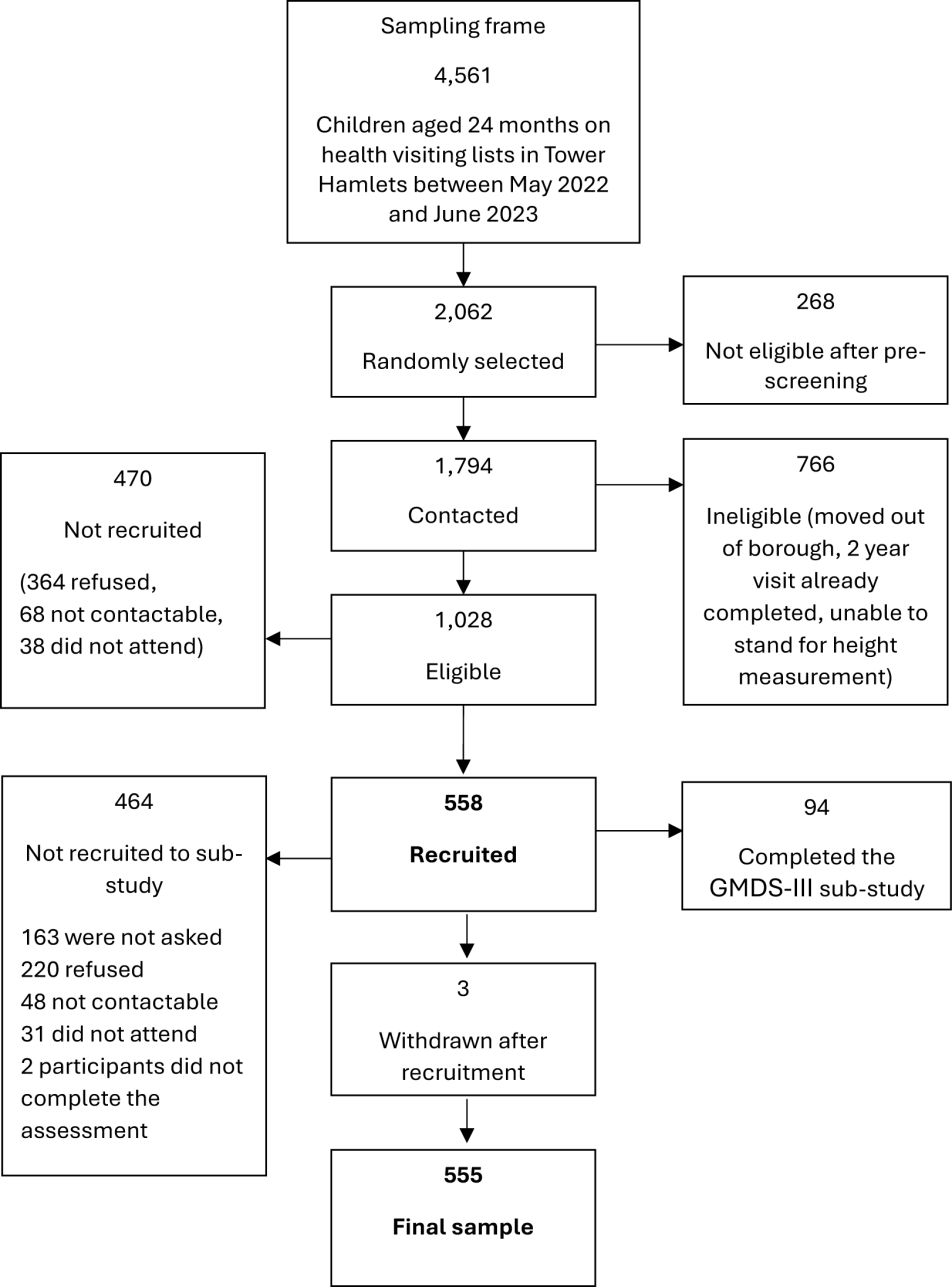
Child Growth Development East London (CGDEL) study sampling flow chart. GMDS III, Griffiths III Mental Development Scales.

Participants were enrolled in the CGDEL study with visits conducted between July 2022 and March 2024. Children aged 2–2.5 years with an HCP visit due were identified through local health visiting service lists. Proportionate random sampling was conducted within four geographic areas in Tower Hamlets (North West, North East, South East and South West), with sample sizes reflecting the number of eligible children in each area. A prescreening system was used to exclude ineligible children, including those unable to stand for accurate height measurement, those outside the eligible age window or those no longer residing in the borough. Eligible families received an invitation letter and a follow-up phone call from the study team. Families who agreed to participate were scheduled for an appointment at a local Children’s Centre with a study HV or NN. Written informed consent was obtained from each child’s primary caregiver. Participating families received a £15 high street voucher to thank them for their time. If a family did not attend, up to two further attempts were made to arrange an alternative date; if the child was still not brought, they were not enrolled in the study. No formal sample size calculation was undertaken; the study size was determined pragmatically by the number of eligible children identified on health visiting lists during the recruitment period and the proportionate random sampling strategy across the four geographic areas, yielding a final analytic sample of 555 children. The full sampling and recruitment process is detailed in figure 1.

### Measures

At the study visit, the HV/NN collected demographic, health and socioeconomic information (see table 1). Sociodemographic data included child sex, age at assessment, ethnicity (categorised as Bangladeshi, White or Other), preterm birth status (<37 weeks’ gestation), maternal education (higher education vs no higher education) and caregiver in receipt of household benefits. Child height was measured to the nearest 0.1 cm using a calibrated portable stadiometer, and weight was measured to the nearest 0.1 kg using a digital stand-on scale, following standardised protocols. Parental heights were measured, but in cases where one parent was not present, their height was estimated by the available parent; where no parental height data were available, DMPH could not be calculated. Child HAZ were calculated using the combined British 1990–WHO growth reference standards.^11^ DMPH, also known as distance to target height, was calculated by combining maternal and paternal heights using a correction for regression to the mean, and then subtracting the standardised target height from the child’s standardised height (see online supplemental methods, section S1, for full details).

**Table 1:** Baseline demographics (above and below ASQ cut-off, All participants)

Measure	Developmental vulnerability, n=141 n (col%, row%)	No developmental vulnerability, n=414 n (col%, row%)	Total, n=555 n (col%)
Age in months*	26 (1.4)	26 (1.4)	26 (1.5)
Sex			
Male	94 (66.7, 33.3)	188 (45.4, 66.7)	282 (50.8)
Female	47 (33.3, 17.2)	226 (54.6, 82.8)	273 (49.2)
Ethnicity (n_s_=140, 413, 553)†			
White any	15 (10.7, 15.8)	80 (19.4, 84.2)	95 (17.2)
Bangladeshi	98 (70.0, 31.8)	210 (50.8, 68.2)	308 (55.7)
Other	27 (19.3, 18.0)	123 (29.8, 82.0)	150 (27.1)
Birth weight in kg (n_s_=138, 404, 542)‡	3.09 (0.56)	3.17 (0.50)	3.15 (0.51)
Gestational age in weeks (n_s_=140, 414, 554)	38.37 (2.00)	38.85 (1.68)	38.73 (1.78)
Gestational age <37 weeks (preterm)	16 (11.3, 43.2)	21 (5.1, 56.8)	37 (6.7)
Any breastfeeding	110 (78.0, 24.7)	336 (81.2, 75.3)	446 (80.4)
Weight in kg (n_s_=132, 399, 531)	12.87 (2.12)	12.62 (1.71)	12.68 (1.82)
Height in cm (n_s_=132, 397, 529)	87.08 (3.57)	87.37 (3.39)	87.30 (3.43)
HAZ <−2 (n_s_=132, 395, 527)§	7 (5.3, 30.4)	16 (4.1, 69.6)	23 (4.4)
DMPH <−2 (n_s_=129, 393, 522)¶	5 (3.9, 41.7)	7 (1.8, 58.3)	12 (2.3)
Mother’s age in years (n_s_=140, 414, 554)	33.08 (5.33)	33.86 (5.11)	33.67 (5.17)
Mother’s education (n_s_=140, 413, 553)			
Higher education	66 (47.1, 20.8)	252 (61.0, 79.2)	318 (57.5)
No higher education	74 (52.9, 31.5)	161 (39.0, 68.5)	235 (42.5)
Benefit status (n_s_=140, 411, 551)			
Receiving benefits	80 (58.3, 28.4)	202 (49.1, 71.6)	282 (51.2)
Not receiving benefits	60 (42.9, 22.3)	209 (50.9, 77.7)	269 (48.8)

* Continuous variables are reported as mean (SD).

† Ethnicity (n_s_=140X, 413Y, 553Z)x

‡ Categorical: Below/Above n (col%, row%); Total n (col%).

§ HAZ <−2 denotes stunting (≥2 SD below the WHO median).

¶ DMPH <−2 denotes being ≥2 SD shorter than expected for genetic potential.

ASQ, Ages and Stages Questionnaire; Col%, non-missing within column; DMPH, deviation from mid-parental height; HAZ, height-for-age Z-score; row%, category total (below+above).

#### Child development

Child development was assessed using the ASQ, Third Edition (ASQ-3),^12^ a widely validated, caregiver-completed developmental screening tool. The ASQ-3 consists of 21 age-specific questionnaires, typically designed in 2-month intervals, covering five developmental domains: communication, gross motor, fine motor, problem solving and personal-social. Each child was assessed using the version that corresponded to their age in months, as per the standard ASQ-3 protocol, thereby ensuring that the milestones assessed were developmentally appropriate. To ensure comprehension, a professional medical translator was present when the caregiver was not fluent in English, providing item-by-item translation and clarification. Each questionnaire contains 30 items scored as ‘Yes’ (10 points), ‘Sometimes’ (5 points) or ‘Not Yet’ (0 points), with higher scores indicating more advanced developmental progress. Total scores for each domain were compared with age-specific standardised thresholds to classify children as ‘on schedule’, ‘close to cut-off’ or ‘below cut-off’, indicating a need for further developmental follow-up. Developmental vulnerability was operationalised as scoring below the ASQ-3 cut-off in ≥1 domain; this is a screening indicator and not a diagnosis. Caregivers completed the ASQ-3 with HVs or NNs, with no adjustments for prematurity applied. The ASQ-3 has demonstrated high internal consistency, interobserver reliability and validity across diverse UK settings, and typically requires about 15 min to complete.^13^

In a nested substudy, a subset of participants (n=94) underwent gold-standard direct developmental assessment using the Griffiths III Mental Development Scales (GMDS-III). During consent for the CGDELstudy, caregivers were asked whether they would be willing to be contacted about the sub-study. Those who volunteered were subsequently contacted by telephone to explain the study procedures. Families who agreed to participate were then invited to a local health clinic, where additional written consent was obtained prior to assessment. For the GMDS-III substudy, only caregivers fluent in English were invited, and assessments were conducted in English as per GMDS-III training guidelines. The GMDS-III provides a detailed, standardised profile across six domains: foundations of learning, language and communication, eye and hand coordination, personal-social-emotional, gross motor and general development. Each domain yields scaled scores, enabling practitioners to identify developmental strengths and areas of concern. The GMDS-III is a modernised revision of the original Griffiths Scales, developed through an international practitioner-informed process to ensure cultural relevance and robust psychometric properties.^14^ Scaled scores were derived for each subdomain using age-normed UK reference data,^15^ with scores ranging from 0 to 13. All assessments were administered by a trained and certified psychologist following standardised procedures.

#### Data management and referral

Data were entered directly into REDCap, a secure online electronic data capture tool, by the HV/NN at the point of collection and were downloaded weekly for monitoring and quality assurance. An in-house growth monitoring algorithm, developed using UK data, was used to flag children with poor growth, based on the child’s standardised height, their height compared with parental heights and an assessment of growth over time. Algorithm details are provided in the online supplemental methods, section S1. Children flagged by the algorithm were referred through the HCP pathway to a specialist child growth clinic at the Royal London Hospital, following local referral protocols.

### Patient and public involvement

We involved parents and health-visiting staff from the same services and cohort via qualitative workshops.^10^ Their priorities and experience informed the research question and refined procedures, including conducting assessments within routine HCP reviews, ASQ-3 administration with translation and feasible appointment scheduling. They did not select outcome measures independently, did not participate in data analysis and did not contribute to authorship decisions.

### Analysis

We first assessed the validity of ASQ-3 cut-off thresholds among children who had both ASQ-3 and GMDS-III assessments undertaken. GMDS-III scaled subdomain scores were compared between children who scored above or below the ASQ cut-off classification using independent sample t-tests (pooled variance). To control for multiple comparisons across the six subdomains, Bonferroni corrections were applied by dividing the overall significance level (0.05) equally among them. Mean differences were reported with 95% CIs and standardised differences (in SD units) were also derived to aid interpretation. In the validation subset (n=94), adjusted associations between HAZ/DMPH (z-scores) and GMDS-III subdomain scaled scores are reported in online supplemental table S4. For GMDS-III, scaled scores are derived from age-normed UK reference data; consequently, age is intrinsically accounted for in the scaled score metric and therefore age was not included in supplementary models.

Next, we conducted two separate binomial logistic regression analyses to examine the association between growth faltering (using HAZ or DMPH as the exposure) and developmental vulnerability, defined as scoring below the ASQ-3 cut-off in at least one developmental domain. For the HAZ model, children were classified as stunted if their HAZ was more than 2 SDs below the WHO reference median. For the DMPH model, children were classified as short for parental height if their DMPH was more than 2 SDs below the pooled participant mean.

For both models, the following covariates were included: standardised child age in months, maternal education (higher education vs no higher education), child sex, caregiver in receipt of household benefits (yes/no) and child ethnicity (Bangladeshi, White or Other). Univariable logistic models and adjusted models using continuous growth metrics are provided in online supplemental tables S2 and S3, respectively. Though the ASQ includes different age-appropriate questionnaires, these windows can span several months, therefore, we included age in months as a covariate in our models. Because missing data were limited, we used complete-case analysis without imputation. Results are presented as ORs with 95% CIs, and statistical significance was defined as p<0.05. Analyses were performed in R (V.4.4.0; R Core Team, 2024).

## Results

### Sample characteristics

A random, area-stratified sample was drawn from health visiting lists in Tower Hamlets (figure 1). Of the 4561 children initially sampled, 2062 (45%) were randomly selected. Following prescreening, 268 children (13%) were deemed ineligible, leaving 1794 suitable for contact. Among these, 1028 (57%) met all inclusion criteria, with 558 (54%) eligible families consenting to participate; 3 subsequently withdrew consent after enrolment, leaving 555 children in the current analysis.

Of 555 children, 141 (25.4%) screened below the ASQ-3 cut-off in ≥1 domain and 414 (74.6%) were above the cut-off for all domains. Among the 141 children who were screened as developmentally vulnerable, 90 (63.8%) were below the cut-off in one domain, 25 (17.7%) in two domains and 26 (18.4%) in three or more domains. The most frequently affected domain was communication (104; 73.8%), followed by personal-social (47; 33.3%), gross motor (32; 22.7%); fine motor (30; 21.3%); problem-solving (28; 19.9%). Baseline characteristics by ASQ-3 status are shown in table 1. Missing child heights and/or missing parental heights meant that HAZ data were available for 527 children, and DMPH data for 522 children.

The cohort included 282 boys (50.8%) and 273 girls (49.2%), with a mean age of 26 months (range: 24–30 months). Over half the children were of Bangladeshi ethnicity, and approximately one-third belonged to other ethnic groups (including mixed). Maternal education levels were diverse: while roughly a quarter of mothers had limited or no formal qualifications, 45% held a university degree.

### Developmental profiles by ASQ cut-off

To explore the robustness of the caregiver-reported ASQ test, we first explored relationships between ASQ scores and directly observed GMDS-III scores in a subgroup of 94 children. Online supplemental table S1 shows the baseline characteristics of participants who undertook the GMDS-III compared with those who did not. We compared GMDS-III subdomain scores between children who fell below any ASQ developmental cut-off and those who did not fall below any cut-off (table 2).

**Table 2:** GMDS-III subdomain scores by ASQ cut-off group

Measure	All participants, n=94	Below ASQ cut-off, n=22	Above ASQ cut-off n=72	Mean Δ*	95% CI, p value
Foundations of learning†	8.69 (3.02)	5.27 (3.25)	9.74 (2.01)	−4.46	−5.60 to −3.32, <0.001
Language and communication	7.11 (4.54)	1.50 (3.33)	8.82 (3.32)	−7.32	−8.93 to −5.71, <0.001
Eye and hand coordination	8.35 (3.65)	4.50 (3.65)	9.53 (2.74)	−5.03	−6.46 to −3.59, <0.001
Personal-social-emotional	7.05 (3.71)	2.27 (2.29)	8.51 (2.69)	−6.24	−7.50 to −4.98, <0.001
Gross-motor	7.83 (3.87)	6.23 (5.92)	8.32 (2.86)	−2.09	−3.92 to −0.26, 0.153
General development	7.45 (3.91)	2.27 (2.49)	9.03 (2.72)	−6.76	−8.05 to −5.46, <0.001

* Mean Δ=mean(Below ASQ cut-off)−mean(Above ASQ cut-off).

† Values are mean scaled scores (SD), n. Final columns show Student’s t-test (pooled variances) with Bonferroni-corrected p values for six comparisons; 95% CIs are unadjusted and presented for effect-size precision.

ASQ, Ages and Stages Questionnaire; GMDS III, Griffiths III Mental Development Scales.

Participants scoring below the ASQ cut-off (n=22) performed significantly worse across every GMDS-III subdomain (except gross motor coordination) compared with those above the cut-off (n=72). The largest standardised differences between groups were seen in foundations of learning (1.90 SD), general development (1.72 SD), eye and hand coordination (1.69 SD) and language and communication (1.68 SD). The difference in personal-social-emotional was also substantial (1.37 SD), while the gross motor domain showed a modest, non-significant difference of 0.54 SD. Together, these findings indicate that children falling below the ASQ cut-off exhibit markedly poorer performance across most GMDS-III subdomains, meaning caregiver-reported developmental vulnerability identified children with significantly lower performance on a battery of direct assessments conducted by a psychologist. These concordant deficits indicate that the ASQ-3 cut-off flags similar developmental difficulties as GMDS-III, supporting criterion validity. We therefore used ASQ-defined developmental vulnerability as our outcome measure for the whole cohort in assessing the relationship between growth faltering and neurodevelopment.

### Predictors of developmental vulnerability

We first assessed the relationship between absolute HAZ and developmental vulnerability, using short stature (HAZ <−2) as the exposure (see table 3).

**Table 3:** Associations between growth measures and ASQ-defined developmental vulnerability: adjusted logistic regression across two models

Variable		HAZ* model			DMPH† model	
	OR	95% CI	P value	OR	95% CI	P value
Growth metric						
HAZ <−2	1.35	(0.47 to 3.49)	0.551	N/A	—	—
DMPH <−2	N/A	—	—	4.18	(1.13 to 14.78)	0.026
Age in months	0.29	(0.13 to 0.66)	0.003	0.28	(0.12 to 0.64)	0.003
Preterm status						
Full-term	Reference			Reference		
Preterm (<37 weeks)	2.53	(1.17 to 5.45)	0.017	2.11	(0.95 to 4.61)	0.061
Maternal education						
No higher education	Reference			Reference		
Higher education	0.72	(0.46 to 1.13)	0.154	0.73	(0.46 to 1.15)	0.173
Child ethnicity						
Bangladeshi	Reference			Reference		
White any	0.45	(0.22 to 0.88)	0.024	0.43	(0.20 to 0.85)	0.020
All other ethnicities	0.57	(0.33 to 0.95)	0.036	0.56	(0.32 to 0.94)	0.031
Child sex						
Male	Reference			Reference		
Female	0.36	(0.23 to 0.56)	<0.001	0.35	(0.22 to 0.55)	<0.001
Benefit status						
Not receiving benefits	Reference			Reference		
Receiving benefits	1.10	(0.70 to 1.72)	0.688	1.20	(0.76 to 1.90)	0.446

* HAZ<−2 denotes stunting >2 SD below the WHO median.

† DMPH<−2 denotes ≥2 SD shorter than expected for genetic potential.

ASQ, Ages and Stages Questionnaire; DMPH, Distance to Mid-Parental Height Z scores; HAZ, Height-for-Age Z scores; N/A, not available.

In the HAZ model, short stature (HAZ <−2 SD) was not significantly associated with increased odds of developmental vulnerability (OR=1.35 (95% CI 0.47, 3.49), p=0.551). However, several other covariates showed relationships with developmental vulnerability. Younger children had higher odds of developmental vulnerability, with every month rise in age associated with a 71% reduced odds of developmental vulnerability (OR=0.29, 95% CI (0.13 to 0.66), p=0.003), while children born preterm had a more than twofold increased risk (OR=2.53, 95% CI (1.17 to 5.45), p=0.017). Girls had significantly lower odds of developmental vulnerability compared with boys (OR=0.36, 95% CI (0.23 to 0.56), p<0.001). Ethnicity was also a strong predictor: compared with the Bangladeshi reference group, white children (OR=0.45, 95% CI (0.22 to 0.88), p=0.024) and those from other minority ethnic backgrounds (OR=0.57, 95% CI (0.33 to 0.95), p=0.036) had lower odds of developmental vulnerability. Maternal education and benefits receipt did not have significant relationships with developmental vulnerability.

In the DMPH model, children who were more than 2 SDs below their mid-parental height had fourfold higher odds of developmental vulnerability (OR=4.18, 95% CI (1.13 to 14.78), p=0.026). The estimate was stable across model checks, and the wide CI reflects the small number of children with DMPH<−2 (N=12 of 522; 2.3%) rather than model instability. As in the HAZ model, younger age (OR=0.28, 95% CI (0.12 to 0.65), p=0.003), female sex (OR=0.35, 95% CI (0.22 to 0.53), p<0.001) and ethnicity remained significant predictors. White children (OR=0.43, 95% CI (0.20 to 0.85), p=0.020) and children from other ethnic backgrounds (OR=0.56, 95% CI (0.32 to 0.94), p=0.031) had reduced odds of developmental vulnerability compared with Bangladeshi children. There was weak evidence that preterm birth was associated with developmental vulnerability (OR=2.11, 95% CI (0.95 to 4.61), p=0.061), while there was no evidence that maternal education (OR=0.73, 95% CI (0.46 to 1.15), p=0.173) and benefit receipt (OR=1.20, 95% CI (0.76 to 1.90), p=0.446) were associated with developmental vulnerability.

Univariable associations are shown in online supplemental table S2. When growth was modelled as continuous z-scores (online supplemental table S3), neither HAZ nor DMPH showed evidence of a linear association with ASQ-defined developmental vulnerability, suggesting any increased risk was concentrated in the extreme tail of the distribution (HAZ<−2 and DMPH<−2). In the GMDS-III validation subset, associations between growth metrics and GMDS-III scaled scores were imprecise (online supplemental table S4), likely reflecting limited power and the small number of children with marked growth faltering in the substudy.

## Discussion

We set out to identify whether screening for linear growth faltering would also identify children at risk of poor neurodevelopment. We found that children with short stature at age 2–2.5 years were not more likely to have developmental vulnerability than children with adequate linear growth. However, children who were short for their genetic potential, based on their distance to parental height, had a fourfold higher risk of having developmental vulnerability (ie, being below a developmental cut-off threshold on the ASQ screening test), compared with children who had normal height based on their genetic potential. These findings underscore the biological significance of growth faltering relative to genetic potential and suggest that using parental heights as part of the routine assessment of child growth could be valuable. Since height is measured through existing contacts with health services in early life, there may be missed opportunities to identify children who are not fulfilling their growth and neurodevelopmental potential. A more formal growth screening programme, which compares child linear growth to parental heights, could therefore plausibly yield dual benefits by identifying children who require further investigation for impaired growth and for neurodevelopmental concerns.

Traditionally viewed as a global health issue, stunting has been under-recognised in HICs. Linear growth faltering is often termed idiopathic short stature when no underlying medical cause is identified, and is then not acted on further. However, recent UK evidence shows that children with short stature are clustered in deprived areas,^8^ and experience persistent cognitive disadvantages throughout childhood, even after adjusting for sociodemographic factors.^9^ Our findings add nuance by demonstrating that children in East London who deviated from their expected genetic height potential were more likely to require additional developmental screening. This supports the idea that growth faltering, particularly when considered relative to parental stature, may serve as a meaningful early marker of developmental vulnerability, at least in urban settings facing multiple overlapping risks.^8 9^ By considering the child’s distance to parental height, it is possible to identify true growth faltering, rather than children with familial short stature, and our data suggest this may be consequential. Linear growth can reflect a constellation of shared risk factors linked to poverty, ethnicity and urban disadvantage.^16 17^ There may also be shared mechanistic pathways, including chronic inflammation, nutritional deficiencies and psychosocial stressors, which may concurrently impact physical growth and neurodevelopment. It is therefore not possible to determine whether our findings are causal or to simply identify a set of shared risk factors or biological pathways. Regardless of the underlying reasons, DMPH is a straightforward, valuable and accurate marker of faltering growth, reflecting cumulative adversity, which may signal early developmental vulnerability, rather than solely medical risk. These children may therefore benefit from further developmental assessment and early support for speech, language and socioemotional difficulties, to improve school readiness. Our results therefore underscore the potential value of integrating developmental considerations into routine growth monitoring.

Through exploring sociodemographic predictors of developmental vulnerability, we found distinct patterns of risk, many of which align with prior literature: male sex, younger age and preterm birth were all associated with poorer developmental outcomes. Notably, children from Bangladeshi backgrounds, who comprised half the cohort in this east London setting, showed the highest rates of developmental vulnerability according to ASQ criteria. However, relatively few Bangladeshi children were identified as being short for parental heights, suggesting that transgenerational factors, such as historically inherited short stature linked to greater rates of adversity might obscure some deviations from genetic potential. This raises important considerations about the appropriateness of universal growth standards for ethnically diverse populations and highlights the potential need for population-specific reference data to avoid underestimating developmental vulnerability in minority groups.

Our study has several strengths, including a substantial sample size and robust representation of an ethnically diverse and socioeconomically disadvantaged urban population, making the findings highly relevant to UK public health policy. Ecological validity is strengthened through integration with the HCP, with all visits conducted by HVs or NNs. Further, the GMDS-III assessment, administered by a trained psychologist, confirmed the validity of caregiver-reported neurodevelopment data, which gives confidence to our findings.

However, several methodological limitations should be acknowledged. The cross-sectional observational design at a single time point limits causal inference and the ability to track developmental trajectories over time. Genetic height potential was estimated using mid-parental height, which may have introduced some measurement error, particularly in cases where one parent’s height was unavailable and had to be estimated. Few children had DMPH below 2 SDs, meaning the precision of our estimates was low, and larger studies across settings are needed to confirm these findings. Missing child and/or parental height data also reduced statistical power. This cohort represented a subsample of the wider study population, which may have introduced bias and reduced the generalisability of findings. Although children were randomly selected, only a subset of those initially sampled contributed to the final analytic cohort after prescreening, eligibility assessment, successful contact, consent and attendance. Though we adjusted models for maternal education and household benefit status, these may not fully capture the social patterning of developmental vulnerability, so residual confounding is likely to remain. Finally, although the ASQ-3 offers important practical advantages at scale and was administered with translator support where needed, it relies on caregiver report and may still be influenced by linguistic, cultural or reporting differences in how developmental items are understood and reported. Ethnicity remained independently associated with developmental vulnerability, raising the possibility of residual confounding from other unmeasured factors. The GMDS-III substudy was included to support criterion validity of ASQ-3 classification rather than to support subgroup inference, and because it was restricted to English-speaking families, it could not fully exclude cross-cultural measurement effects. Furthermore, the GMDS-III validation substudy was also not fully representative of the main cohort, particularly with respect to ethnicity, with lower participation among Bangladeshi families. This may limit the generalisability of the validation findings and mean that cross-cultural measurement effects cannot be fully excluded. These considerations mean that between-group comparisons should be interpreted cautiously and underscore the need for longitudinal follow-up using culturally sensitive assessment approaches.

Our findings highlight important avenues for future research and public health policy. This study contributes to growing evidence that growth trajectories and developmental outcomes are closely intertwined, even in HICs, particularly among children living in deprivation. By framing growth as an early signal of developmental vulnerability rather than simply a marker of underlying medical disorders, we advocate for integration of physical and cognitive monitoring within routine child health surveillance, by considering the value of height screening for identifying children with other underlying vulnerabilities. Longitudinal studies incorporating behavioural assessments, biomarker measurements and neuroimaging methodologies would help clarify the mechanisms linking early growth faltering with developmental outcomes to inform targeted interventions. Evaluating the predictive validity of DMPH across diverse populations and settings will be crucial to determine its potential integration into existing surveillance frameworks, such as the HCP and the National Child Measurement Programme, which currently focuses on obesity but does not identify linear growth faltering. Expanding growth monitoring to include genetic potential alongside traditional height-for-age assessments may enhance the early identification of children at risk of developmental vulnerability. This could provide a scalable strategy to identify developmental vulnerabilities early and enable pre-school enrichment programmes to be targeted to those in need, to improve school readiness.

## Supplementary material

10.1136/bmjph-2025-004567 online supplemental file 1

10.1136/bmjph-2025-004567 online supplemental table 1

## Data Availability

Data are available on reasonable request.
