# Supplementary material for: Identifying developmental vulnerability through linear growth screening: a UK cross-sectional study
Source: BMJ Public Health. 2026 Jun 23;4(2):e004567. doi: 10.1136/bmjph-2025-004567 (PMC13295896; doi:10.1136/bmjph-2025-004567)
Supplement: online supplemental file 1 [file bmjph-4-2-s001.docx]

# Online Supplementary Methods (S1)

# Computation of height SDS (HAZ/HSDS), target height, and DMPH

# S1.1 Scope

This supplement details how child height standard deviation scores (HSDS/HAZ) were computed from UK–WHO references, how target (mid-parental) height was derived with a regression-to-the-mean adjustment, and how Deviation from Mid-Parental Height (DMPH) was calculated and used as a study exposure.

# S1.2 Reference samples and eligibility (for formula development)

For developing and checking the target‑height adjustment, cohort data from the Millennium Cohort Study (MCS) and Born in Bradford (BiB) were used. Eligibility for these derivations required at least one non‑outlier height (−4 to +4 SDS) between 24–101 months (BiB) or 32–97 months (MCS), singleton births, and no growth‑affecting conditions (diagnoses listed in Table S1). Children <24 months were excluded (supine length differs from standing height). For DMPH calculations that use parental heights, two‑parent heights were preferred; a validated one‑parent correction is provided below.

# S1.3 Height SDS (HAZ/HSDS)

**Reference:** UK–WHO growth standards.

**Software:** Stata (zanthro) or equivalent to compute z‑scores.

**Definition:**

HSDS ≡ HAZ = (child height − μ_ref(age, sex)) / σ_ref(age, sex)

# S1.4 Target (mid-parental) height and DMPH

## S1.4.1 Notation and rationale

We define MH_SDS and FH_SDS as the mother’s and father’s adult height SDS (using an appropriate adult UK height reference with LMS parameters). Target height incorporates a regression‑to‑the‑mean adjustment, so the predicted child height lies closer to the population mean than a simple parental average.

## S1.4.2 Selected target-height formula (two-parent case)

An adapted Wright & Cheetham‑type regression performed best in UK data:

TH_SDS = 0.1 + 0.7 × ((MH_SDS + FH_SDS) / 2)

Performance (summary): In MCS, mean distance to target at ~17y (adult height) was −0.00 (SD 0.79); 61% were within 0.67 SDS and 91% within 1.33 SDS. At 7–8y, mean 0.05 (SD 0.90); 54% and 86% within 0.67 and 1.33 SDS, respectively. BiB ages 7–8y corroborated the choice of this formula.

## S1.4.3 One-parent correction (when one parental height is missing)

Derived from MCS regressions and validated in BiB:

TH_SDS = 0.1 + 0.4 × ParentHeight_SDS

This outperformed using an unadjusted single parent and an alternative published correction.

## S1.4.4 Computing DMPH

DMPH = HSDS − TH_SDS

Interpretation: negative = shorter than genetic expectation; positive = taller. For this manuscript, DMPH was standardised using the mean and SD from the analytic cohort (pooled across age and sex), and dichotomised as DMPH < −2 SD.

# S1.5 Worked example (two-parent case)

1) Convert parental heights to adult height SDS (MH_SDS, FH_SDS) using an adult UK reference with LMS parameters.

2) Compute target height: TH_SDS = 0.1 + 0.7 × ((MH_SDS + FH_SDS) / 2).

3) Compute child HSDS/HAZ from UK–WHO references.

4) DMPH = HSDS − TH_SDS.

(For one‑parent case, use the 0.4 multiplier formula in step 2.)

# Tables

## Table S1. Exclusion diagnoses used for formula development (illustrative)

| Category | Codes/examples |
| --- | --- |
| Syndromic causes of short stature | e.g., Turner syndrome, trisomies (ICD‑10: Q9x) |
| Endocrine disorders | e.g., growth hormone deficiency, hypothyroidism (E23.0, E03.x) |
| Chronic systemic disease | e.g., chronic kidney disease, congenital heart disease (N18.x, Q20–Q28) |
| Skeletal dysplasias | e.g., achondroplasia (Q77.x) |

## Table S2. Performance summary of the selected target-height formula

| Dataset / age | Mean (DMPH) | SD (DMPH) | % within 0.67 SD | % within 1.33 SD |
| --- | --- | --- | --- | --- |
| MCS, ~17y | −0.00 | 0.79 | 61% | 91% |
| MCS, 7–8y | 0.05 | 0.90 | 54% | 86% |
| BiB, 7–8y | Best performance replicated | — | — | — |

# S1.6 Notes on terminology

We use DMPH consistently in the manuscript; some literature refers to the same construct as distance to target height (DTH).

# S1.7 Selected references for S1

Wright CM, Cheetham TD. The strengths and limitations of parental heights as a predictor of attained height. Arch Dis Child. 1999;81(3):257–60.

Hermanussen M, Cole J. The calculation of target height reconsidered. Horm Res. 2003;59(4):180–3.
