# Supplementary material for: Identifying developmental vulnerability through linear growth screening: a UK cross-sectional study
Source: BMJ Public Health. 2026 Jun 23;4(2):e004567. doi: 10.1136/bmjph-2025-004567 (PMC13295896; doi:10.1136/bmjph-2025-004567)
Supplement: online supplemental table 1 [file bmjph-4-2-s002.docx]

**Appendices – Supplementary Tables**

Supplementary Table S1. Characteristics of children who completed the GMDS sub-study.

| Measure | | GMDS-III completed, n = 94 n (col%, row%) | No GMDS-III completed, n = 461 n (col%, row%) | Total, n = 555 n (col%) |
| --- | --- | --- | --- | --- |
| Age in months^a^ | | 27 (1.2) | 26 (1.5) | 26 (1.4) |
| Sex | |  |  |  |
| Male | | 52 (55.3%, 18.4%) | 230 (49.9%, 81.6%) | 282 (50.8%) |
| Female | | 42 (44.7%, 15.4%) | 231 (50.1%, 84.6%) | 273 (49.2%) |
| Ethnicity (ns = 94, 459, 553)^b^ | |  |  |  |
| White Any | | 27 (28.7%, 28.4%) | 68 (14.8%, 71.6%) | 95 (17.2%) |
| Bangladeshi | | 34 (36.2%, 11.0%) | 274 (59.7%, 89.0%) | 308 (55.7%) |
| Other | | 33 (35.1%, 22.0%) | 117 (25.5%, 78.0%) | 150 (27.1%) |
| Birthweight in kg (ns = 91, 451, 542)^c^ | | 3.11 (0.53) | 3.16 (0.51) | 3.15 (0.51) |
| Gestational age in weeks (ns = 94, 460, 554) | | 38.62 (2.37) | 38.75 (1.64) | 38.73 (1.78) |
| Gestational age <37 weeks (preterm) | | 8 (8.5%, 21.6%) | 29 (6.3%, 78.4%) | 37 (6.7%) |
| Any breastfeeding | | 75 (79.8%, 16.8%) | 371 (80.5%, 83.2%) | 446 (80.4%) |
| Weight in kg (ns = 91, 440, 531) | | 12.94 (1.84) | 12.63 (1.81) | 12.68 (1.82) |
| Height in cm (ns = 88, 441, 529) | | 87.47 (3.45) | 87.27 (3.43) | 87.30 (3.43) |
| HAZ < -2 (ns = 87, 440, 527)^d^ | | 6 (6.9%, 26.1%) | 17 (3.9%, 73.9%) | 23 (4.4%) |
| DMPH < -2 (ns = 86, 436, 522)^e^ | | 3 (3.5%, 25.0%) | 9 (2.1%, 75.0%) | 12 (2.3%) |
| Mother’s age in years (ns = 94, 460, 554) | | 34.69 (5.40) | 33.46 (5.10) | 33.67 (5.17) |
| Mother's education (ns = 93, 460, 553) | |  |  |  |
| Higher education | | 61 (65.6%, 19.2%) | 257 (55.9%, 80.8%) | 318 (57.5%) |
| No higher education | | 32 (34.4%, 13.6%) | 203 (44.1%, 86.4%) | 235 (42.5%) |
| Benefit status (ns = 94, 457, 551) | |  |  |  |
| Receiving benefits | | 38 (40.4%, 13.5%) | 244 (53.4%, 86.5%) | 282 (51.2%) |
| Not receiving benefits | | 56 (59.6%, 20.8%) | 213 (46.6%, 79.2%) | 269 (48.8%) |
| Developmental screening | |  |  |  |
| ASQ-3 developmental vulnerability identified | | 22 (23.4%, 15.6%) | 119 (25.8%, 84.4%) | 141 (25.4%) |
| a Continuous variables are reported as mean (SD). | | | |  |
| b (x, y, z) represents n in each group when missing data is present. | | | |  |
| c Categorical: Below/Above n (col%, row%); Total n (col%). Col% = non-missing within column; row% = category total (Below + Above). | | | |  |
| d HAZ < −2 denotes stunting (≥ 2 SD below the WHO median). | | | |  |
| e DMPH < −2 denotes being ≥ 2 SD shorter than expected for genetic potential. | | | |  |

Supplementary Table S2. Univariable logistic regression for ASQ-defined developmental vulnerability (below cut-off in ≥1 domain).

| Variable |  | OR | 95% CI, | *p* | N |  |  |  |
| --- | --- | --- | --- | --- | --- | --- | --- | --- |
| Linear growth metric |  |  |  |  |  |  |  |  |
| < 2 HAZ ^a^ |  | 1.33 | [0.50, 3.18] | 0.672 | 527 |  |  |  |
| < 2 DMPH ^b^ |  | 2.22 | [0.65, 7.09] | 0.543 | 522 |  |  |  |
| Age in months |  | 0.32 | [0.15, 0.66] | 0.002 | 555 |  |  |  |
| Preterm status |  |  |  |  |  |  |  |  |
| *Full-term* |  | Reference | | | |  |  |  |
| *Preterm (<37 weeks)* |  | 2.40 | [1.20, 4.72] | 0.012 | 555 |  |  |  |
| Maternal Education |  |  |  |  |  |  |  |  |
| *No Higher Education* |  | Reference | | | |  |  |  |
| *Higher Education* |  | 0.57 | [0.39, 0.84] | 0.004 | 553 |  |  |  |
| Child Ethnicity |  |  |  |  |  |  |  |  |
| *Bangladeshi* |  | Reference | | | |  |  |  |
| *White Any* |  | 0.40 | [0.21, 0.71] | 0.003 | 553 |  |  |  |
| *All other Ethnicities* |  | 0.47 | [0.29, 0.75] | 0.002 | 553 |  |  |  |
| Child Sex |  |  |  |  |  |  |  |  |
| *Male* |  | Reference | | | |  |  |  |
| *Female* |  | 0.42 | [0.28, 0.62] | < 0.001 | 555 |  |  |  |
| Benefit Status |  |  |  |  |  |  |  |  |
| *Not receiving Benefits* |  | Reference | | | |  |  |  |
| *Receiving Benefits* |  | 1.38 | [0.94, 2.04] | 0.103 | 551 |  |  |  |

Note. OR = Odds Ratio; CI = Confidence Interval

^a^ HAZ: Height-for-Age Z scores; HAZ<−2 denotes stunting >2 SD below the WHO median.

^b^ DMPH: Distance to Mid-Parental Height Z scores; DMPH<−2 denotes ≥2 SD shorter than expected for genetic potential.

Supplementary Table S3. Associations between continuous growth measures and ASQ-defined developmental vulnerability: adjusted logistic regression across two models.

| Variable |  |  | HAZ^a^ Model |  |  |  | DMPH^b^ Model | |  |
| --- | --- | --- | --- | --- | --- | --- | --- | --- | --- |
|  |  | OR | 95% CI, | *p* |  | OR | 95% CI, | *p* | |
| Linear growth metric |  |  |  |  |  |  |  |  | |
| *HAZ* |  | 0.92 | [0.74, 1.15] | 0.456 |  | N/A | — | — | |
| *DMPH* |  | N/A | — | — |  | 0.94 | [0.75, 1.18] | 0.619 | |
| Age in months |  | 0.28 | [0.12, 1.15] | 0.003 |  | 0.28 | [0.12, 0.64] | 0.003 | |
| Preterm status |  |  |  |  |  |  |  |  | |
| *Full-term* |  | Reference | | |  | Reference | | | |
| *Preterm (<37 weeks)* |  | 2.51 | [1.16, 5.40] | 0.018 |  | 2.19 | [0.98, 4.82] | 0.051 | |
| Maternal Education |  |  |  |  |  |  |  |  | |
| *No Higher Education* |  | Reference | | |  | Reference | | | |
| *Higher Education* |  | 0.73 | [0.46, 1.14] | 0.164 |  | 0.73 | [0.46, 1.15] | 0.173 | |
| Child Ethnicity |  |  |  |  |  |  |  |  | |
| *Bangladeshi* |  | Reference | | |  | Reference | | | |
| *White Any* |  | 0.46 | [0.22, 0.90] | 0.029 |  | 0.46 | [0.21, 0.91] | 0.032 | |
| *All other Ethnicities* |  | 0.59 | [0.34, 0.99] | 0.049 |  | 0.57 | [0.33, 0.96] | 0.039 | |
| Child Sex |  |  |  |  |  |  |  |  | |
| *Male* |  | Reference | | |  | Reference | | | |
| *Female* |  | 0.36 | [0.23, 0.56] | < 0.001 |  | 0.36 | [0.23, 0.56] | < 0.001 | |
| Benefit Status |  |  |  |  |  |  |  |  | |
| *Not receiving Benefits* |  | Reference | | |  | Reference | | | |
| *Receiving Benefits* |  | 1.11 | [0.71, 1.74] | 0.660 |  | 1.18 | [0.75, 1.87] | 0.478 | |

Note. OR = Odds Ratio; CI = Confidence Interval.

*^a^ HAZ: height-for-age z-score (WHO); HAZ (z-score).*

*^b^ DMPH: distance to mid-parental height (z-score).*

Supplementary Table S4. Associations between growth measures and GMDS-III scaled scores in the validation subset (n=94): adjusted linear regression by domain across two models.

| Variable |  |  | HAZ^a^ Model |  |  |  | DMPH^b^ Model |  |
| --- | --- | --- | --- | --- | --- | --- | --- | --- |
|  |  | OR | 95% CI, | *p* |  | OR | 95% CI, | *p* |
| Growth metric |  |  |  |  |  |  |  |  |
| *HAZ < −2* |  | 0.34 | [0.07, 1.49] | 0.163 |  | N/A | — | — |
| *DMPH < −2* |  | N/A | — | — |  | 0.60 | [0.06, 6.11] | 0.668 |
| Preterm status |  |  |  |  |  |  |  |  |
| *Full-term* |  | Reference | | |  | Reference | | |
| *Preterm (<37 weeks)* |  | 0.63 | [0.16, 2.49] | 0.511 |  | 0.52 | [0.12, 2.12] | 0.364 |
| Maternal Education |  |  |  |  |  |  |  |  |
| *No Higher Education* |  | Reference | | |  | Reference | | |
| *Higher Education* |  | 1.46 | [0.58, 3.65] | 0.420 |  | 1.53 | [0.61,  3.86] | 0.372 |
| Child Ethnicity |  |  |  |  |  |  |  |  |
| *Bangladeshi* |  | Reference | | |  | Reference | | |
| *White Any* |  | 6.50 | [2.23, 19.92] | 0.001 |  | 7.67 | [2.55, 24.41] | < 0.001 |
| *All other Ethnicities* |  | 0.59 | [1.84 ,12.06] | 0.002 |  | 4.58 | [1.79, 12.07] | 0.003 |
| Child Sex |  |  |  |  |  |  |  |  |
| *Male* |  | Reference | | |  | Reference | | |
| *Female* |  | 1.87 | [0.86, 2.10] | 0.120 |  | 1.79 | [0.82, 3.98] | 0.152 |
| Benefit Status |  |  |  |  |  |  |  |  |
| *Not receiving Benefits* |  | Reference | | |  | Reference | | |
| *Receiving Benefits* |  | 0.83 | [0.33, 2.10] | 0.689 |  | 0.98 | [0.37, 2.60] | 0.971 |
|  |  |  |  |  |  |  |  |  |

Note. OR = Odds Ratio; CI = Confidence Interval.
